# An Internet-Delivered Cognitive Behavioral Therapy for Depression and Anxiety Among Clients Referred and Funded by Insurance Companies Compared With Those Who Are Publicly Funded: Longitudinal Observational Study

**DOI:** 10.2196/16005

**Published:** 2020-02-04

**Authors:** Heather D Hadjistavropoulos, Vanessa Peynenburg, Swati Mehta, Kelly Adlam, Marcie Nugent, Kirsten M Gullickson, Nickolai Titov, Blake Dear

**Affiliations:** 1 Department of Psychology University of Regina Regina, SK Canada; 2 Department of Physical Medicine and Rehabilitation Western University London, ON Canada; 3 Online Therapy Unit University of Regina Regina, SK Canada; 4 MindSpot Department of Psychology Macquarie University Sydney Australia

**Keywords:** internet, disability, depression, anxiety, insurance, cognitive behavior therapy

## Abstract

**Background:**

Anxiety and depression are leading causes of disability but are often undertreated. Internet-delivered cognitive behavioral therapy (ICBT) improves access to treatment by overcoming barriers to obtaining care. ICBT has been found to be efficacious in research trials and routine care, but there is limited research of ICBT when it is recommended and funded by insurance companies for clients on or recently in receipt of disability benefits or accommodations.

**Objective:**

The aim of this study was to examine ICBT engagement, treatment satisfaction, and effectiveness among individuals involved with 2 insurance companies. The 2 samples were benchmarked against published outcomes from a publicly funded (PF) ICBT clinic.

**Methods:**

Individuals who were on or recently in receipt of disability benefits and were either insurance company (IC) employees (n=21) or IC plan members (n=19) were referred to ICBT funded by the respective insurance companies. Outcomes were benchmarked against outcomes of ICBT obtained in a PF ICBT clinic, with clients in the clinic divided into those who reported no involvement with insurance companies (n=414) and those who were on short-term disability (n=44). All clients received the same 8-week, therapist-assisted, transdiagnostic ICBT course targeting anxiety and depression. Engagement was assessed using completion rates, log-ins, and emails exchanged. Treatment satisfaction was assessed posttreatment. Depression, anxiety, and disability measures were administered pretreatment, posttreatment, and at 3 months.

**Results:**

All samples showed high levels of ICBT engagement and treatment satisfaction. IC employees experienced significant improvement at posttreatment (depression *d*=0.77; anxiety *d*=1.13; and disability *d*=0.91) with outcomes maintained at 3 months. IC plan members, who notably had greater pretreatment disability than the other samples, experienced significant moderate effects at posttreatment (depression *d*=0.58; anxiety *d*=0.54; and disability *d*=0.60), but gains were not maintained at 3 months. Effect sizes at posttreatment in both IC samples were significantly smaller than in the PF sample who reported no insurance benefits (depression *d*=1.14 and anxiety *d*=1.30) and the PF sample who reported having short-term disability benefits (depression *d*=0.95 and anxiety *d*=1.07). No difference was seen in effect sizes among IC employees and the PF samples on disability. However, IC plan members experienced significantly smaller effects on disability *d*=0.60) compared with the PF sample with no disability benefits *d*=0.90) and those on short-term disability benefits *d*=0.94).

**Conclusions:**

Many clients referred and funded by insurance companies were engaged with ICBT and found it acceptable and effective. Results, however, were not maintained among those with very high levels of pretreatment disability. Small sample sizes in the IC groups are a limitation. Directions for research related to ICBT funded by insurance companies have been described.

## Introduction

### Background

Anxiety and depression are highly prevalent in adult populations and are often associated with a high degree of disability [1]. According to the World Health Organization, depression is the leading cause of ill health and disability worldwide [2], while anxiety disorders have been identified as the 9th leading cause of disability worldwide [3]. These disorders come with high personal and economic costs. The Mental Health Commission of Canada estimates that mental health concerns cost the Canadian economy approximately Can $50 billion annually [4]. Almost Can $6 billion of this is attributed to lost productivity from absenteeism, presenteeism, and turnover in working adults. Moreover, 30% of disability or insurance claims are attributed to mental health concerns. Thus, there is significant pressure to identify and implement effective interventions to assist individuals with mental health concerns in relieving their symptoms and improving work-related disability.

Although there is great need for mental health treatment, several barriers exist that prevent individuals with mental health concerns from accessing effective treatment, such as concerns about mental health stigma, time constraints, and rural or remote geographical locations [5]. Internet-delivered cognitive behavioral therapy (ICBT) has received increased attention in clinical trials and routine care because it overcomes these barriers to care [6]. ICBT typically consists of weekly Web-based modules that provide psychoeducation and skills about managing symptoms of anxiety and depression [7]. Often, therapist assistance is offered in the form of weekly secure emails or telephone calls from a therapist. ICBT has been shown to be as effective as face-to-face cognitive behavioral therapy (CBT) with comparable drop-out rates [8]. In fact, organizations such as Health Quality Ontario have reviewed the growing evidence for the efficacy of ICBT and recommended that therapist-assisted ICBT for individuals with mild-to-moderate symptoms of anxiety or depression be publicly funded (PF) [9].

Despite the growing evidence base supporting the use of ICBT for depression and anxiety, additional research is necessary to illustrate the effectiveness of ICBT when offered in different contexts and populations. Replication trials essentially serve to establish the ecological validity or generalizability of efficacious interventions delivered under different circumstances or to different populations [10]. In Canada, mental health care is funded in a variety of ways. Canada has a PF health care system that provides all Canadian residents with access to medically necessary hospital and physician services through federal taxes that are transferred to the provinces and territories [11]. Each of the 13 provinces and territories are then required to have their own health care insurance plans to ensure that residents do not pay for hospital or physician services out-of-pocket. With this federal funding, provinces and territories also typically cover some mental health care services in PF settings (eg, hospital, community mental health clinics, and Web-based clinics). Some residents of Canada also have access to additional mental health care through insurance company (IC) plans paid for by employers. Alternatively, some residents may pay for some mental health services out of pocket.

To date, in Canada, there has been reported effectiveness of PF ICBT [12], but there are no published trials in Canada on the effectiveness of ICBT among clients who are involved with an IC owing to their mental health symptoms (eg, currently or recently in receipt of disability benefits or receiving workplace accommodations), especially when ICBT is recommended and funded by the IC.

### Outcomes in Insurance Company Clients

Research on face-to-face CBT indicates that outcomes of CBT may not be as promising for IC clients, suggesting it would be valuable to explore if this is also the case with ICBT. Specifically, in a recent meta-analysis conducted by Salomonsson et al [13], the efficacy of CBT for individuals on disability leave for mental health concerns was examined, and effect sizes for sick leave and reduction in symptoms were significant, but quite small (Hedges *g*=0.17 and 0.21, respectively). In comparison, the most recent review of CBT for anxiety and depression in the general adult population found larger effect sizes, even when effect sizes were adjusted for publication bias (*g*=0.59 and 0.65, respectively) [14].

Theoretically, there are multiple factors that could contribute to poorer outcomes among individuals on disability leave involved with an IC [15]. Models of mental health recognize that mental health is influenced by individuals (eg, symptoms), social (eg, work stress), and environmental (eg, access to service and injustice) factors [15]. At the individual level, those involved with ICs may have greater symptom severity, which has been associated with poorer treatment outcomes [16]. Specifically, ICs often have requirements for the severity or duration of symptoms before a disability claim can be granted, especially long-term disability, which may contribute to more severe symptoms in IC groups [17]. In terms of social factors, individuals involved with an IC may have poorer outcomes than those not involved with an IC as a result of challenges they face when returning to work after being on disability leave, such as challenges meeting workload responsibilities [18] or being faced with lack of support [19,20]. In terms of environmental factors, it has been found that the length of approval times for claims, especially for long-term disability, can create a delay in treatment, which could negatively impact treatment outcomes [21]. It is also possible that being involved with an IC represents a negative or stressful experience for clients which then influences treatment outcomes [22]. Past research suggests, for instance, that mental health claims are regarded as more complex for ICs to manage because of diagnostic challenges as well as stigma associated with mental health issues [23]. Past research also suggests that claims management can significantly impact mental health [22,24,25] and that individuals with mental health conditions are significantly more likely to have negative experiences with disability claims and return to work [26].

### Aims of the Study

The aim of this study was to examine the generalizability of outcomes of ICBT by assessing engagement, treatment satisfaction, and effectiveness of ICBT in 2 IC samples. In both samples, clients were receiving or were recently in receipt of disability benefits or workplace accommodations for mental health symptoms, and the ICs referred clients to and funded ICBT. Results from these 2 IC samples were benchmarked against a previously published trial that established the effectiveness of the same ICBT program when it was offered to clients who sought ICBT from a PF ICBT clinic [12]. Benchmarking is an established method for comparing outcomes in different groups and has been used previously in studies on the effectiveness of CBT [27,28]. Extrapolating from past face-to-face CBT research [13], we expected that IC plan members and IC employees would exhibit significant but smaller improvements on symptom measures than PF clients. Other comparisons were considered exploratory in nature given limited past research. The results of this study have implications for the use of ICBT funded by ICs; if results are promising, ICs may have greater interest in referring to and funding ICBT, which has the potential to not only improve client well-being but reduce substantial costs associated with mental health concerns.

## Methods

### Study Design and Ethics

This study followed an observational pre-post test design with a 3-month follow-up. Research ethics board approval was obtained from the University of Regina for the study of all samples. The PF ICBT trial was registered (ISRCTN42729166), and the results have previously been published but not divided by whether clients were or were not in receipt of short-term disability benefits [12].

One sample consisted of IC plan members, while the second sample consisted of IC employees. The 2 samples were examined separately as there was a requirement to report mid-treatment and posttreatment outcomes to a case worker for the IC plan members following disability benefit guidelines. Separate examination of the 2 samples also provided opportunity for comparison of background characteristics, which revealed some differences between the IC samples. Results from these 2 IC samples were benchmarked against a previously published trial of the same ICBT program when it was offered to clients who sought ICBT from a PF ICBT clinic [12]. The PF sample was subdivided into those who reported no insurance benefits and those who reported being on short-term disability benefits. In the latter case, although clients reported short-term disability benefits, there was no contact between the PF clinic and the clients’ insurance provider. All samples received the same 8-week transdiagnostic ICBT program that addressed symptoms of both anxiety and depression. Benchmarking is a well-known strategy for examining outcomes in situations where random assignment to groups is not feasible [29].

Across all samples, to assess engagement, we examined the number of log-ins, number of emails exchanged between clients and therapists, and percentage of clients who completed 4 out of 5 ICBT lessons that covered the primary treatment strategies. To assess effectiveness, we examined improvements in depression, anxiety, and disability at posttreatment and 3-month follow-up. To assess treatment satisfaction, we examined ratings of ICBT posttreatment. Furthermore, in the 2 IC samples, we examined qualitative feedback related to strengths and challenges of ICBT.

### Clients

Clients were recruited during the following time periods: IC employees (September 2017 to May 2018), IC plan members (June 2017 to June 2018), and both PF samples (November 2013 to July 2015). In all samples, ICBT was delivered by the same clinic, but the service was either funded by the government or the IC. IC employees were in receipt of, or had recently been in receipt of, short-term or long-term disability payments or had mental health workplace accommodations or benefits while at work. Recruitment for IC employees was through an email invitation sent by the insurer to eligible employees. Interested IC employees voluntarily visited the website to enroll in ICBT. IC plan members had an open short- or long-term disability claim related to anxiety or depression or were in receipt of mental health accommodations or benefits at work. Recruitment for IC plan members involved case managers providing plan members with information about ICBT, first through a phone call and then an email link to the ICBT website. With client consent, case managers of the IC plan members were sent reports on client outcomes at mid- and posttreatment. As described in a previously published study [12], all PF clients self-referred to ICBT after learning about ICBT through community mental health clinics (167/458, 36.5%), family physicians (99/458, 21.7%), word of mouth (68/458, 14.8%), media (56/458, 12.2%), Web searches and email announcements (54/458, 11.8%), or printed advertisements (14/478, 2.9%). The PF short-term disability clients self-reported being in receipt of short-term disability benefits, but their care was PF and the ICBT clinic had no contact with their IC.

To be included in the study, clients from all samples completed a Web-based screening followed by telephone screening to assess their eligibility for ICBT. All clients had to meet the following criteria: 18 years of age or older; residents of Saskatchewan (all samples) or Ontario (IC employees and IC plan members only); endorse at least mild symptoms of anxiety or depression; access to the internet and comfortable using computers; and willing to provide a health care professional as an emergency contact. Exclusion criteria included the following: reporting symptoms of mania, psychosis, posttraumatic stress disorder, alcohol, or substance misuse that were not being effectively managed; high risk for suicide based on plan or intent in the last year; or hospitalization in the last year related to suicide risk or severe mental health concerns.

For the IC employees, 24 individuals completed the screening process, 88% (21/24) were accepted and began ICBT, 71% (15/21) completed posttreatment measures, and 57% (12/21) completed 3-month follow-up measures. For the IC plan members, 23 completed the screening process, 83% (19/23) were accepted and began ICBT, 84% (16/19) completed posttreatment measures, and 58% (11/19) completed 3-month follow-up measures. For the PF sample, 545 completed the screening process, 76.0% (414/545) were accepted and began ICBT, 81.9% (339/414) completed posttreatment measures, and 75.1% (311/414) completed 3-month follow-up measures. For the PF short-term disability sample, 65 clients completed the screening process, 68% (44/65) were accepted and began ICBT, 91% (40/44) completed posttreatment measures, and 66% (29/44) completed 3-month follow-up measures.

### Intervention

All clients received the same 8-week transdiagnostic ICBT course (*Wellbeing Course*) that addresses both anxiety and depression. The course was developed by the eCentre Clinic at Macquarie University in Sydney, Australia [30], and is licensed for use by the Online Therapy Unit [12]. The course contains 5 lessons that focus on the following: (1) the cognitive behavioral model and symptom identification; (2) thought monitoring and challenging; (3) dearousal strategies and pleasant activity scheduling; (4) graduated exposure; and (5) relapse prevention. Lessons are available in a slideshow format with downloadable materials and weekly homework assignments to facilitate skill acquisition. Clients also have access to client stories and extra resources as needed (eg, communication, problem solving, and sleep).

### Therapists

All IC employees and IC plan members were assigned to 1 Web-based therapist employed by the ICBT clinic who had experience in ICBT and possessed a Master’s degree in Social Work. All PF clients [12] were assigned to a therapist who worked directly in the ICBT clinic (n=2 registered psychologists; n=1 registered social worker; n=13 psychology graduate students; and n=9 social work graduate students) or in 1 of 8 community mental health clinics associated with the clinic (n=10 registered psychologists; n=25 registered social workers; n=5 registered nurses; and n=1 registered counselor). All therapists participated in a 1-day workshop [12] before delivering ICBT. Graduate students received supervision from a registered provider. A more in-depth description of the training of these therapists is available elsewhere [12].

### Therapist Support

Most of the contact between therapists and clients occurred over a secure Web-based messaging system. Clients were encouraged to email their therapist throughout the week as they reviewed treatment materials; the therapist, on the contrary, checked in with clients and responded to emails by secure email on 1 predesignated day each week. Telephone calls were made in the following circumstances: clients had not logged into the website in the past week, clients were not responding to emails, clients requested a phone call, or therapists were concerned about client safety because of an increase in depression symptoms or suicidal ideation as assessed by questionnaires.

### Outcome Measures

Clients completed measures at pretreatment, posttreatment, and 3-month follow-up. The measures were completed by participants on the same website that was used to deliver the intervention. Participants received reminder emails to complete measures at posttreatment and 3-month follow-up. Measures of anxiety and depression were also administered at the beginning of lessons 2 to 5 to allow therapists to monitor symptoms.

#### Patient Health Questionnaire 9-Item

The Patient Health Questionnaire-9 (PHQ-9) [31] is a 9-item validated self-report questionnaire that is used to assess depression symptom severity. Total scores range from 0 to 27 with scores being interpreted as indicative of mild (5-9), moderate (10-14), moderately severe (15-19), and severe (20-27) depressive symptoms [32]. A cut-off score of 10 or higher is used to identify those who are likely to have a diagnosis of depression [20]. The PHQ-9 has good psychometric properties [31]. The Cronbach alpha in this study was .85.

#### Generalized Anxiety Disorder 7-item

The Generalized Anxiety Disorder (GAD)-7 [33] is a 7-item validated self-report questionnaire that is used to assess anxiety symptom severity, with total scores ranging from 0 to 21. Total scores are interpreted as indicative of mild (5-9), moderate (10-14), and severe (15-21) anxiety symptoms [24]. A cut-off score of 10 or higher is used to identify those who are likely to have a Diagnostic and Statistical Manual of Mental Disorders 5th edition diagnosis [33]. The generalized anxiety disorder 7-item (GAD-7) has strong psychometric properties [33]. The Cronbach alpha in this study was .88.

#### Sheehan Disability Scale

The Sheehan Disability Scale (SDS) [34] is a 3-item validated measure of functional impairment in work/school, social life, and family life. Scores range from 0 to 30, with higher scores indicating higher levels of impairment. The SDS has high internal consistency and sensitivity to treatment and has been used in previous ICBT research [35]. The Cronbach alpha in this study was .84.

#### Engagement

Engagement was measured by assessing the percentage of clients who completed the course, number of emails sent to therapist, and number of log-ins to the course.

#### Treatment Satisfaction

At the end of treatment, clients were asked if they felt that the treatment was worth their time (*Yes* or *No*) and if they would recommend the course to a friend (*Yes* or *No*). Moreover, clients were asked to rate treatment satisfaction (response options included *very dissatisfied*, *dissatisfied*, *neutral*, *satisfied*, and *very satisfied*), whether participating in the course affected their confidence in managing their symptoms and whether the course increased their motivation to seek help in the future if needed (response options for the last 2 questions were *greatly reduced*, *reduced*, *no change*, *increased*, and *greatly increased*). IC plan members and IC employees also answered 2 open-ended questions to obtain feedback on the most helpful elements of ICBT and suggestions for improvement.

### Statistical Analysis

Data were analyzed using SPSS 23 (IBM). To begin, descriptive statistics were used to describe and compare samples in terms of demographics and scores on pretreatment depression, anxiety, and disability. To assess engagement, we compared samples on the percentage of clients who completed 4 out of 5 core lessons over 8 weeks, the mean number of log-ins, and the mean number of emails exchanged between clients and therapists. Group differences were analyzed using one-way analyses of variance for continuous variables and chi-square tests for categorical variables. When tests were significant, post hoc analyses were conducted to examine group differences.

When examining outcome measures, missing data were imputed using multiple imputation based on chained equations [36]. The imputation model included demographic variables such as age and symptom severity at baseline as predictors. A total of 40 imputations were generated to avoid producing a large Monte Carlo error [37]. Pooled data were used for the analysis [38]. To begin, as we had not previously analyzed IC data, we examined the IC samples using generalized estimation equation (GEE) modeling to evaluate effectiveness of treatment in the 2 IC groups [39]. An unstructured working correlation matrix and maximum likelihood estimation were used. A gamma distribution with a log link response scale was specified to address positive skewness and proportionally changing scores in the dependent variables [40]. Pairwise comparisons were used to examine the statistical significance of changes in the outcomes examining group and time effects.

Additional statistics were calculated for benchmarking purposes. Cohen *d* effect sizes and 95% confidence intervals were calculated for the within-group effects based on the estimated marginal mean values derived from the GEE analysis. Consistent with the literature, *d*=0.20 was regarded as a small effect, *d*=0.50, a medium effect, and *d*=0.80, a large effect [41]. Effect size difference of 0.20 or greater from the benchmark groups (PF clients and PF short-term disability clients) were considered to be clinically significant [41]. In addition, consistent with the literature, we interpreted a within-group effect size of *d*=0.24 as the minimally important difference [42]. To assist with understanding effect sizes, we also calculated the average percentage change and 95% confidence intervals across time for each outcome measure from the GEE analyses. The percentages of clients reporting improvements in symptoms of 30% and deterioration of 30% from pre- to posttreatment and pretreatment to follow-up were calculated and compared among the groups using chi-square tests; 30% was selected as an additional method for identifying at least some meaningful improvement on measures [43]. When tests were significant, post hoc analyses were conducted to examine group differences.

To assess treatment satisfaction, we compared groups using chi-square tests in terms of the percentage of clients who found the course helpful, the percentage who would recommend the course to a friend, and the percentage who reported being *very satisfied* or *satisfied* with treatment, having *greatly increased* or *increased* confidence in managing symptoms, and *greatly increased* or *increased* motivation to seek additional health care in the future. As above, when tests were significant, post hoc analyses were conducted to examine group differences.

Among the IC plan members and IC employees, to analyze qualitative feedback on the most helpful elements of ICBT and suggestions for improving ICBT, we used conventional content analysis [44] to identify themes in clients’ responses to 2 open-ended questions.

## Results

### Client Characteristics

The mean age of the clients ranged from 38.92 to 45.95 years. The majority of clients in all 4 groups were female (range: 32/44, 73%-17/21, 81%), had more than a high school education (range: 35/44, 80%-18/21, 86%), were married or common law (range: 11/19, 58%-33/44, 75%), and Caucasian (range: 16/19, 84%-75/414, 93.1%). A large proportion of clients lived in a small city or rural area (range: 194/414, 48.8%-11/19, 58%). Among IC employees, 29% (6/21) were at work with mental health accommodations or benefits, 48% (10/21) were on short-term disability, and 24% (5/21) were on long-term disability. Among IC plan members, 16% (3/19) were at work with mental health accommodations or benefits, 32% (6/19) were on short-term disability, and 53% (10/19) were on long-term disability. Examination of group differences revealed no differences in terms of sex, marital status, education, ethnicity, or location; however, differences among groups in terms of age and employment status were found. Post hoc analyses showed PF clients were significantly younger than PF short-term disability clients (*P<*.01) and IC plan members (*P*=.01) but comparable to IC employees (*P*=.11). No significant difference in age was seen among IC plan members, IC employees, and PF short-term disability clients. Table 1 includes additional demographic information for the clients, separated by sample.

Significant differences were seen among the groups on pretreatment measures of depression (PHQ-9, *F*_3,489_*=*9.78; *P=.*01), anxiety (GAD-7, *F*_3,489_*=*5.16; *P*<.01), and disability (SDS, *F*_3,489_*=*17.55; *P*<.01). See Table 2 for mean scores. Post hoc analyses examining pretreatment PHQ-9 scores showed that IC plan members had significantly higher scores compared with the PF clients (mean difference=5.61; *P*<.01) and PF short-term disability clients (mean difference=3.73; *P*=.02), but not IC employees (mean difference=1.75; *P*=.33). IC employees had significantly higher pretreatment PHQ-9 scores compared with PF clients (mean difference=3.86; *P*<.01) but not PF short-term disability clients (mean difference=1.98; *P*=.18). PF clients had significantly lower scores compared with PF short-term disability clients (mean difference=1.88; *P*=.04).

**Table 1:** Demographic characteristics of clients separated by sample.

Sample		IC^a^ employees (n=21)		IC plan members (n=19)		PF^b^ short-term disability clients (n=44)		PF clients (n=414)		Statistical significance					
										*F* test (3,489)		χ^2^ (*df*)		*P* value	
**Age (years)**											**7.84**		**—^c^**		**.01**
	Mean (SD)		42.76 (9.42)		45.47 (11.96)		46.36 (11.05)		38.26 (12.63)						
	Range		28-65		23-63		24-64		18-74						
**Sex, n (%)**											**—**		**0.6 (3)^d^**		**.89**
	Male		4 (19)		5 (27)		12 (27)		108 (26.7)						
	Female		17 (81)		14 (74)		32 (73)		296 (73.3)						
**Marital status, n (%)**											**—**		**0.6 (3)^d^**		**.24**
	Married/common law		15 (71)		11 (58)		33 (75)		248 (61.1)						
	Unmarried		6 (29)		8 (42)		11 (25)		158 (38.9)						
**Education, n (%)**											**—**		**0.5 (3)^e^**		**.93**
	High school diploma or less		3 (14)		3 (16)		9 (21)		75 (18.5)						
	Greater than high school^f^		18 (86)		16 (84)		35 (80)		331 (81.5)						
**Employment status, n (%)**											**—**		**617.7 (9)^e^**		**<.01**
	Working		6 (29)		3 (16)		—		282 (69.5)						
	Unemployed/student/retired/not reported		—		—		—		124 (30.5)						
	Short-term disability		10 (48)		6 (32)		44 (100)		—				—		
	Long-term disability		5 (24)		10 (53)		—		—				—		
**Ethnicity, n (%)**											**—**		**2.4 (3)^g^**		.49
	Caucasian		19 (91)		16 (84)		37 (90)		375 (93.1)						
	Non-Caucasian or not reported		2 (10)		3 (16)		4 (10)		28 (6.9)						
**Location, n (%)**											**—**		**2.4 (3)^e^**		**.49**
	Large city (over 200, 000)		9 (43)		8 (42)		19 (43)		212 (51.2)						
	Small center		12 (57)		11 (58)		25 (57)		194 (47.8)						

^a^IC: insurance company.

^b^PF: publicly funded.

^c^Not applicable.

^d^N=488.

^e^N=490.

^f^Some college or university education.

^g^N=484.

**Table 2:** Estimated marginal means and 95% CI for primary outcomes separated by sample.

Estimates		Estimated marginal means		
		Pretreatment mean (95% CI)	Posttreatment mean (95% CI)	3-month follow-up mean (95% CI)
**PHQ-9^a^**				
	IC^b^ employees (N=21)	16.10 (13.51-18.68)	11.04 (8.14-13.95)	11.09 (8.70-13.49)
	IC plan members (N=19)	17.84 (16.15-19.53)	14.60 (11.58-17.61)	17.94 (14.50-21.38)
	PF^c^ short-term disability clients (N=44)	14.11 (12.61-15.61)	8.32 (6.30-10.34)	6.88 (5.14-8.62)
	PF clients (N=406)	12.23 (11.68-12.78)	5.81 (5.27-6.34)	5.72 (5.17-6.27)
**GAD-7^d^**				
	IC employees (N=21)	13.67 (11.82-15.52)	8.07 (5.79-10.36)	9.69 (7.43-11.95)
	IC plan members (N=19)	15.53 (13.47-17.59)	12.72 (10.25-15.18)	15.41 (13.13-17.69)
	PF short-term disability clients (N=44)	13.11 (11.31-14.92)	6.66 (4.94-8.37)	6.07 (4.90-7.24)
	PF clients (N=406)	11.57 (11.07-12.08)	5.14 (4.68-5.59)	5.18 (4.71-5.65)
**SDS^e^**				
	IC employees (N=21)	24.10 (22.00-26.19)	16.93 (12.78-21.08)	17.83 (14.50-21.16)
	IC plan members (N=19)	25.68 (24.13-27.24)	21.59 (17.70-25.48)	23.33 (19.00-27.67)
	PF short-term disability clients (N=44)	22.43 (20.20-24.67)	13.96 (10.96-16.95)	12.29 (19.64-14.95)
	PF clients (N=404)	17.22 (16.47-17.96)	9.83 (8.99-10.68)	8.96 (8.11-9.82)

^a^PHQ-9: Patient Health Questionnaire-9.

^b^IC: insurance company.

^c^PF: publicly funded.

^d^GAD-7: Generalized Anxiety Disorder-7.

^e^SDS: Sheehan disability scale.

On the pretreatment GAD-7 scores, IC plan members had significantly higher scores compared with the PF clients (mean difference=3.95; *P*<.01) but not the PF short-term disability clients (mean difference=2.41; *P*=.09) or IC employees (mean difference=1.86; *P*=.26). IC employees did not differ significantly on GAD-7 from the other 3 groups (*P* range=.07 to .69). PF clients and PF short-term disability clients did not differ significantly (*P*=.06). On pretreatment SDS scores, PF clients had significantly lower disability scores compared with the other 3 groups, who did not differ from each other.

### Engagement

Overall, there was a high level of engagement in ICBT among clients in all 4 samples. There were no differences among groups in the percentage of clients who completed 4 out of 5 lessons (15/21, 71% IC employees; 17/19, 90% IC plan members; 346/414, 83.5% PF clients; and 38/44, 86% PF short-term disability clients; χ^2^_3_,_N=490_=3.0 *P*=.40). There were also no differences in the mean number of times clients logged into the program (IC employees mean 18.76, SD 9.82; IC plan members mean 24.05, SD 12.49; PF clients mean 22.60, SD 13.81; PF short-term disability clients mean 24.59, SD 12.32; *F*_3,489_*=*0.955; *P*=.41) or the mean number of emails clients sent to their therapists (IC employees mean 3.71, SD 3.44; IC plan members mean 3.84, SD 3.45; PF clients mean 4.69, SD 4.01; PF short-term disability clients mean 4.93, SD 3.22; F_3,489_=0.759; *P*=.51).

### Treatment Effects for Insurance Company Employees and Insurance Company Plan Members

The means and 95% confidence intervals of primary outcome measures are reported in Table 2. The GEE analyses indicated significant effects for Time on symptoms of depression (Wald’s χ^2^_2,N=1470_=100.8; *P*<.001), anxiety (Wald χ^2^_2,N=1470_=122.1; *P*<.001), and disability (Wald χ^2^_2,N=1470_=131.9; *P*<.001). Pairwise comparisons found significant improvements in scores from pretreatment to posttreatment (*P*<.01) but not pretreatment to follow-up (*P*=.97) for SDS scores. Significant improvement in PHQ-9 and GAD-7 scores were revealed from pretreatment to posttreatment and pretreatment to follow-up (range *P*=.04 to <.01). Main effects of Group were seen on the PHQ-9 (Wald χ^2^_3,N=1470_=79.0; *P*<.001), GAD-7 (Wald χ^2^_3,N=1470_=74.7; *P*<.001), and SDS (Wald χ^2^_3,N=1470_=88.3; *P*<.001) showing that the IC plan members had overall higher scores than IC employees. Time by Group interactions were also observed on all the primary outcome measures (PHQ-9, Wald χ^2^_6,N=1470_=23.8, *P*<.001; GAD-7, Wald χ^2^_6,N=1470_=27.6, *P*<.001; and SDS, Wald χ^2^_6,N=1470_=14.4, *P*=.02).

### Benchmarking

Table 3 provides Cohen d and 95% confidence interval values from pretreatment to posttreatment and to 3-month follow-up for the 4 samples. From pre- to posttreatment on the PHQ-9, effects of IC plan members were inferior to those of IC employees (IC employees *d*=0.77 and IC plan members *d*=0.58). The IC plan members were also significantly inferior to the PF short-term disability clients *d*=0.95) and PF clients *d*=1.14). IC employees were inferior to PF clients but not PF short-term disability clients. From pre- to posttreatment on the GAD-7, IC plan members had significantly inferior effect sizes *d*=0.54) compared with IC employees *d*=1.13), PF short-term disability clients *d*=1.07), and PF clients *d*=1.30); the IC employees did not differ significantly from the PF short-term disability clients or PF clients. On the SDS pre- to posttreatment, IC plan members had a significantly inferior effect size *d*=0.60) compared with the other 3 groups, which did not differ from each other (IC employees *d*=0.91; PF short-term disability clients *d*=0.94; and PF clients *d*=0.90). From pretreatment to 3-month follow-up, on measures of depression, anxiety, and disability, IC employees had large effect sizes (range *d*=0.80 to 0.94) that were significantly better than the IC plan members (range *d*=0.02 to 0.32) but inferior to the 2 benchmarking samples (range *d*=1.00 to 1.35).

Consistent with recommendations in the literature [42], within-group effect sizes of *d*=0.24 were determined as being a minimally important difference. All effect sizes from pretreatment to posttreatment on all measures in all samples were regarded as meeting this threshold. On the contrary, from pretreatment to 3-month follow-up, IC plan members were not found to have an effect size large enough to meet the minimally important difference threshold.

**Table 3:** Clinical reliable change from pretreatment to posttreatment and 3-month follow-up separated by group.

Estimates		Effect sizes from pretreatment, Cohen *d* (95% CI)		Improvement≥30%		Deterioration≥30%	
		To posttreatment	To 3-month follow-up	Pre- to posttreatment (%)	Pre- to 3-month follow-up (%)	Pre- to posttreatment (%)	Pre- to 3-month follow-up (%)
**PHQ-9^a^**							
	IC^b^ employees	0.77 (0.13 to 1.38)	0.84 (0.19 to 1.45)	48	50	0	14
	IC plan members	0.58 (-0.08 to 1.22)	-0.02 (-0.65 to 0.62)	32	156	11	37
	PF^c^ short-term disability clients	0.95 (0.50 to 1.38)	1.30 (0.83 to 1.75)	67	74	7	2
	PF clients	1.14 (0.99 to 1.29)	1.14 (0.99 to 1.20)	75.9	75.9	2.7	5.0
**GAD-7 ^d^**							
	IC employees	1.13 (0.45 to 1.75)	0.80 (0.16 to 1.42)	62	38	5	5
	IC plan members	0.54 (-0.12 to 1.18)	0.02 (-0.61 to 0.66)	26	16	5	26
	PF short-term disability clients	1.07 (0.62 to 1.51)	1.35 (0.88 to 1.81)	72.7	74	5	2
	PF clients	1.30 (1.15 to 1.45)	1.30 (1.15 to 1.45)	78.3	75.9	3.2	3.2
**SDS^e^**							
	IC employees	0.91 (0.26 to 1.53)	0.94 (0.29 to 1.56)	38	43	0	5
	IC plan members	0.60 (-0.06 to 1.24)	0.32 (-0.33 to 0.95)	26.3	21	0	11
	PF short-term disability clients	0.94 (0.49 to 1.37)	1.21 (0.74 to 1.65)	51.2	64	2	0
	PF clients	0.90 (.76 to 1.05)	1.00 (0.85 to 1.15)	65.3	67.8	7.0	7.2

^a^PHQ-9: Patient Health Questionnaire-9.

^b^IC: insurance company.

^c^PF: publicly funded.

^d^GAD-7: Generalized Anxiety Disorder-7.

^e^SDS: Sheehan disability scale.

To further facilitate interpretation of the effects, Table 3 also includes descriptive information about the improvement in symptoms of 30% as well as deterioration of 30% for each group for each measure from pre- to posttreatment and from pretreatment to 3-month follow-up.

On the posttreatment PHQ-9, a greater proportion of clients experienced 30% reduction in scores among the PF clients (314/414, 75.9%) and PF short-term disability clients (30/44, 67%) compared with both IC employees (10/21, 48%) and IC plan members (6/19, 32%; χ^2^_3,N=485_=25.1; *P*=.001). The same significant pattern was found at 3-month follow-up (χ^2^_3,N=485_=38.07; *P*<.001).

On posttreatment GAD-7 scores, a significantly lower proportion of IC plan members (5/19, 26%) experienced 30% reduction in GAD-7 scores compared with IC employees (13/21, 62%), PF short-term disability clients (32/44, 73%), and PF clients (324/414, 78.3%), while no significant differences were seen among IC employees and the 2 benchmarking groups (χ^2^_3,N=489_=28.4; *P*=.001). At 3-month follow-up, a significantly lower proportion of IC employees (8/21, 38%) and IC plan members (3/19, 16%) reported 30% reduction in GAD-7 scores compared with the PF short-term disability clients (33/44, 75%) and PF clients (314/414, 75.8%; χ^2^_3,N=485_=44.6; *P*<.001).

On posttreatment SDS scores, IC employees (8/21, 38%) and IC plan members (5/19, 26%) did not differ significantly from PF short-term disability clients (23/44, 51%) in proportion of individuals experiencing 30% reduction on SDS scores. Furthermore, IC employees had a similar proportion of individuals experiencing 30% reduction on SDS scores compared with PF short-term disability clients. However, significantly lower proportions of individuals experiencing 30% reduction on SDS scores were seen between IC plan members and IC employees and PF clients (χ^2^_3,N=481_=19.2; *P*<.001). A similar pattern was seen among the groups at 3-month follow-up on 30% reduction on SDS scores (χ^2^_3,N=484_=21.81; *P*<.001).

In terms of deterioration of 30%, no significant differences were seen among the groups at posttreatment on PHQ-9 scores (χ^2^_3,N=487_=6.0; *P*=.11), GAD-7 scores (χ^2^_3,N=489_=0.5; *P*=.92), or SDS scores (χ^2^_3,N=485_=4.3; *P*=.23) and at 3-month follow-up on SDS scores (χ^2^_3,N=485_=4.0; *P*=.27). However, at 3-month follow-up on PHQ-9 scores, results showed a greater proportion of IC plan members (7/19, 37%), and IC employees (3/21, 14%) experienced deterioration compared with PF clients (21/414, 5.0%) and PF short-term disability clients (1/44, 2%; χ^2^_3,N=488_=34.5; *P*<.001). Similarly, at 3-month follow-up on the GAD-7, significant differences in deterioration were seen among IC plan members (5/19 26%) compared with IC employees (1/21, 5%), PF clients (13/414, 3.2%), and PF short-term disability clients (1/44, 2%; χ^2^_3,N=488_=25.0; *P*<.001).

### Treatment Satisfaction

There were no differences among clients on any of the measures of treatment satisfaction. Nearly all clients stated they were confident in recommending the program to a friend (IC employees: 21/21, 100%; IC plan members: 19/19, 100%; PF short-term disability clients: 43/44, 98%; and PF clients: 392/414, 94.7%; χ^2^_3,N=387_=2.1; *P*=.56) and the program was worth their time (IC employees: 21/21, 100%; IC plan members: 19/19, 100%; PF short-term disability clients: 42/44, 95%; and PF clients: 393/414, 95.0%; χ^2^_3,N=386_=1.4; *P*=.70). Most clients reported that they were *satisfied* or *very satisfied* with ICBT (IC employees: 19/21, 91%; IC plan members: 13/19, 68%; PF short-term disability clients: 36/44, 82%; and PF clients: 355/414, 85.7%; χ^2^_3,N=490_=5.1, *P*=.17). Clients reported that the program *increased* or *greatly increased* their confidence in managing symptoms (IC employees: 17/21, 81%; IC plan members: 16/19, 84%; PF short-term disability clients: 16/19, 86%; and PF clients: 390/414, 94.3%; χ^2^_3,N=490_=10.5; *P*=.02) as well as their motivation to seek additional help in the future (IC employees: 19/21, 91%; IC plan members: 16/19, 84%; PF short-term disability clients: 31/44, 71%; and PF clients: 353/414, 85.2%; χ^2^_3,N=490_=1.5; *P*=.68).

### Client Feedback

A total of 14 IC employees and 13 IC plan members provided feedback on the most helpful elements of ICBT as well as suggestions for improvement. There was variability in what employees found most helpful. Half of the IC employees (7/14, 50%) found the lesson on controlled breathing and activity planning to be most helpful, while 36% (5/14) identified the lesson on thought challenging as the most helpful and 21% (3/14) identified the lesson on graduated exposure as the most helpful. The majority of IC plan members preferred the lesson on thought challenging (8/13, 61%), while 23% (3/13) preferred the lesson on controlled breathing and activity planning and 1 found the lesson on graded exposure (1/13, 8%) to be most helpful. One IC plan member (1/13, 8%) said that *nothing* was helpful in the course.

In terms of improvements, 29% (4/14) of IC employee clients reported that they would not change anything about the course, while 36% (5/14) of the IC employees made suggestions about lesson content (eg, more psychoeducation and more client stories) and course layout or aesthetics (eg, font color, bookmarking function in lessons, and audio on slides). Other suggestions made by single clients included the use of fewer surveys, allowing more time for lesson completion, and placing less pressure on clients to complete lessons; 3 23% (3/13) of the IC plan members stated that they would not change anything about ICBT; 15% (2/13) of the IC plan members found the client stories difficult to relate to and 1 client (1/13, 8%) suggested increasing the number of client stories. One IC plan member felt the pace of the course was too fast (1/13, 8%) and one found the lessons were too long (1/13, 8%). An additional recommendation was that therapist support should be increased (3/13, 23%).

## Discussion

### Principal Findings

Therapist-assisted ICBT is a promising alternative to face-to-face CBT that increases client access to care. There is limited research, however, on outcomes of ICBT among clients who are insurer-referred and -funded. It is important to study ICBT under these circumstances as there is growing interest among insurance companies in funding ICBT, but little evidence to draw on to inform the potential engagement, treatment satisfaction, and effectiveness of ICBT.

In this study, we examined the effectiveness of ICBT for anxiety and depression among individuals who were on short-term or long-term disability or had mental health accommodations or benefits while working and were either IC employees or IC plan members. These 2 samples were benchmarked to PF clients and PF short-term disability clients. All 4 groups reported improvements on measures of depression, anxiety, and disability at posttreatment (see Table 3). All of the groups, except IC plan members, maintained improvement on measures of depression, anxiety, and disability when examining effects from pretreatment to 3-month follow-up (see Table 3).

When examined in terms of 30% improvement in scores, there was a substantial number of clients who experienced improvements at posttreatment and 3-month follow-up in each group, although the pattern overall suggested improvements were best in PF clients and lowest in IC plan members, both at posttreatment and at 3-month follow-up. For example, 26% (5/19) to 32% (6/19) of IC plan members, 38% (8/21) to 62% (13/21) of IC employees, 51% (23/44) to 73% (32/44) of PF short-term disability clients, and 65.0% (269/414) to 78% (333/414) of PF clients experienced 30% improvement on at least one of the measures at posttreatment. At 3-month follow-up, 16% (3/19) to 21% (4/19) of IC plan members, 38% (8/21) to 50% (11/21) of IC employees, 63% (28/44) to 75% (33/44) of PF short-term disability clients, and 68% (282/414) to 76% (314/414) of PF clients experienced 30% improvement on one of the measures at 3-month follow-up. It was encouraging that deterioration of 30% was low and not significantly different among the samples at posttreatment on depression, anxiety, or disability scores, or at 3-month follow-up on disability scores. Nevertheless, at 3-month follow-up on depression, results showed a greater proportion of IC plan members (7/19, 37%) and IC employees (3/21, 14%) experienced 30% deterioration compared with PF clients (21/414, 5.0%) and PF short-term disability clients (1/44, 2%; χ^2^_3_,_N=488_=34.5; *P*<.001). At 3-month follow-up on the anxiety, a greater number of IC plan members (5/19, 26%) had 30% deterioration compared with the other samples where deterioration ranged from 2% (1/44) to 5.0% (21/414).

Consistent with the face-to-face literature [13], overall, effect sizes were lower in IC employees and IC plan members than the benchmarking samples at posttreatment and at 3-month follow-up. This was particularly striking among IC plan members who did not maintain gains at 3-month follow-up. Of note, IC plan members had significantly higher scores on depression, anxiety, and disability than PF clients, greater depression, and disability scores than PF short-term disability clients, and greater disability scores than IC employees. The finding that IC plan members had poorer outcomes is consistent with past research on the impact of severity of conditions on ICBT outcomes [45]. Previous research has suggested that while individuals with severe symptoms of depression can benefit from ICBT, they often require longer treatment and may benefit from using ICBT in addition to other services [45]. Some studies exclude clients with severe depression [45] based on the rationale that these clients require more clinician contact and a longer duration of treatment. In this study, it is possible that IC plan members could have benefitted from receiving ICBT either for longer periods or as an adjunct to face-to-face care. Of note, this is consistent with qualitative feedback provided by some of these clients.

The other interesting finding to emerge from the analysis was that PF short-term disability clients, for the most part, had better outcomes than IC clients (eg, at both posttreatment and 3-month follow-up, effect sizes for PF short-term disability clients were better for depression, anxiety, and disability than both IC samples, with the exception that disability was comparable to IC employees at posttreatment). Nevertheless, PF short-term disability outcomes were not quite as strong as the PF sample that reported no use of insurance benefits (eg, effect sizes were lower on depression and anxiety but not disability at both posttreatment and 3-month follow-up). It is possible that PF short-term disability clients had better outcomes than IC plan members because they had lower scores on depression, anxiety, and disability, but it is not clear why the PF short-term disability clients did better than IC employees since their pretreatment scores were similar. Future research should elucidate what might account for why those receiving short-term disability appear to do better when ICBT is PF rather than insurer funded, and whether this relates to factors such as motivation or confidence in treatment or concerns that treatment outcome may be communicated with the insurer and impact benefits.

Despite lower effect sizes than the benchmarking samples and the less favorable outcomes for IC plan members, especially at 3-month follow-up, there was a comparable level of engagement and treatment satisfaction among the 4 groups. It is particularly noteworthy that IC plan members, who had smaller improvements and outcomes that were not maintained at 3-month follow-up, still regarded ICBT as worth their time (100%) and that they would recommend the course to a friend (100%). The majority also reported that their confidence in managing symptoms either *increased* or *greatly increased* (13/19, 68%), and that their motivation to seek additional help in the future if they needed either *increased* or *greatly increased* (16/19, 84%).

The findings of this research had a subsequent impact on the insurance companies involved in the research. Both companies perceived the results as positive and have secured contracts with private companies who now provide ICBT to their clients. A strength of this study was the inclusion of qualitative comments from clients. With some clients reporting greatest benefit from thought challenging, others indicating controlled breathing and activity planning and others graduated exposure, the feedback highlighted that clients differ significantly in terms of what skills they find beneficial, thus emphasizing the importance of providing clients with multiple skills during treatment. Similarly, there is considerable diversity in suggestions for improving ICBT, ranging from desire for more stories to improvements in course layout or aesthetics (eg, font color, bookmarking function in lessons, and audio on slides) to improvements in delivery method (eg, time for lesson completion and support). The suggestions provide direction for improvement but also highlight that needs of clients vary.

### Limitations

Despite these strengths, there were several limitations that impacted the conclusions that can be drawn from this study. Both IC samples had small sample sizes (*n*=21 and 19), and caution should be taken when generalizing these study results. A significant amount of follow-up data was missing from IC employees (9/21, 43%) and IC plan members (8/19, 42%) at 3-month follow-up, which makes it difficult to draw conclusions about the effects of ICBT at 3-month follow-up. It should also be noted that in this study, both of the IC samples were assigned to 1 specific therapist and that the analytical models utilized cannot account for possible therapist effects. Furthermore, we do not have information on whether our samples differed in terms of socioeconomic status or diagnostic status. Information on actual time using the website or completing specific pages on the website or suggested homework was not collected, which could provide valuable information about client engagement. Although the IC plan members seemed to benefit less from the ICBT course, this conclusion is made solely on their symptoms and their self-reported benefits. An objective measure of the benefit of the ICBT would be to assess the number of sick days or return to work following completion of the intervention.

### Future Directions

The findings of this study provide directions for future research. In particular, among those referred and funded by IC, it would be valuable to compare ICBT with other forms of treatment within a randomized controlled trial. With larger samples, it would be valuable to compare the outcomes of ICBT among clients who were at work with accommodations and benefits compared with those who were on short-term disability and long-term disability. Obtaining additional outcomes beyond self-report would also be beneficial, such as health care utilization, absenteeism, and presenteeism. The qualitative comments suggest ways in which the ICBT course may be modified to better meet the needs of clients involved with an IC, such as including more personal stories relevant to clients and potentially providing more time to complete the course or more therapist support. Future trials could compare weekly to twice weekly contact with a therapist or examine the possibility of using ICBT as an adjunct to face-to-face services or providing greater attention to return to work as has been done in face-to-face CBT [13]. Some past research suggests that outcomes of CBT can be improved by including a return to work intervention among individuals on or at risk of being on short- or long-term disability. For example, at 12-month follow-up, participants who underwent work-focused CBT had significantly higher levels of work participation (44.2% vs 37.2%), with the difference remaining significant at 18-month follow-up. Furthermore, participants in the work-focused CBT group experienced a significant reduction in symptoms of anxiety and depression, as well as an increase in health-related quality of life. Of note, the recruitment with the insurance companies took a significant period of time, suggesting that more attention needs to be given to increasing the knowledge and pretreatment expectations of ICBT in this population. Past research suggests that even a brief 5-min video increases interest in ICBT [46]. Now that the insurance companies have secured contracts with private companies for delivering ICBT to their clients, it would be beneficial to examine the outcomes of ICBT offered by these companies. It is unknown how comparable programs are in terms of content, delivery methods, and ultimately outcomes. In addition, in the future, it would be beneficial to examine barriers and facilitators to implementation of ICBT when funded by insurance companies [47].

### Conclusions

This study contributes to the existing literature regarding ICBT and highlights the engagement, treatment satisfaction, and effectiveness of ICBT among individuals involved with insurance companies as a result of depression and anxiety. To our knowledge, this is the first benchmarking study to compare the effectiveness of ICBT among clients who are employees of an IC and clients who have an open claim with an IC, compared with clients seeking PF ICBT in routine care. It contributes to the literature on ICBT for individuals with more severe symptoms [45], such as those who have an open disability case with an IC. The findings highlight potential directions for improving outcomes among clients insurer-referred and funded.

## Abbreviations

CBT: cognitive behavioral therapy
GAD-7: generalized anxiety disorder 7-item
GEE: generalized estimation equation
IC: insurance company
ICBT: internet-delivered cognitive behavioral therapy
PF: publicly funded
PHQ-9: patient health questionnaire-9
SDS: Sheehan disability scale
